# An Interpersonal Perspective on HR Attributions: Examining the Role of Line Managers, Coworkers, and Similarity in Work-Related Motivations

**DOI:** 10.3389/fpsyg.2019.01509

**Published:** 2019-07-02

**Authors:** Susanne Beijer, Karina Van De Voorde, Maria Tims

**Affiliations:** ^1^School of Business and Economics, Vrije Universiteit Amsterdam, Amsterdam, Netherlands; ^2^Department of Human Resource Studies, Tilburg University, Tilburg, Netherlands

**Keywords:** HR attributions, interpersonal perspective, line manager, worker, motivation, strategic HRM, multi-level modeling

## Abstract

Given that various studies have linked Human Resource (HR) attributions to important individual and organizational outcomes, the question that arises is what causes these HR attributions. By taking an interpersonal perspective it is examined how employees both individually as well as collectively interpret HR practices. Based on social information processing theory this study among 87 line manager–employee–coworker triads shows that line managers affect HR attributions of employees, and that employees also mutually influence each other’s HR attributions. This mutual influence process between coworkers is strengthened by similarity in work-related motivations. Our findings support the proposition that employees’ social environment at work, particularly their line manager and coworker, matters in HR attribution processes. This stresses the importance of considering the social environment at work to more fully understand the factors that shape employees’ understandings of HR practices.

## Introduction

A substantial body of research has focused on understanding the relationship between Human Resource (HR) practices and employee and organizational outcomes (e.g., Jiang et al., 2012; Van de Voorde et al., 2012; Guest, 2017). As part of the attempt to gain a better understanding of the mechanisms through which HR practices affect outcomes, there has been a growing focus on how employees attribute meanings to the HR practices that are adopted in the organization (e.g., Guest, 1999; Bowen and Ostroff, 2004; Purcell and Hutchinson, 2007; Nishii and Wright, 2008). An important stream of research in this respect is focused on the relationship between HR attributions and attitudinal and behavioral outcomes (Nishii et al., 2008; Fontinha et al., 2012; Hewett et al., 2018). HR attributions reflect why employees think that certain HR practices are implemented in their work unit (Nishii et al., 2008) and have been shown to be related to important employee attitudes and behaviors (e.g., Nishii et al., 2008). Given this, the question that arises is what causes these HR attributions, or put differently, what the predictors of these HR attributions are. To date, such research is lacking. A better understanding of the predictors of HR attributions will help to advance knowledge about how employees (individually and/or collectively) come to understand what the organization expects, values, and rewards (Bowen and Ostroff, 2004). Moreover, this knowledge will inform managers regarding how Human Resource Management (HRM) perceptions can be managed.

To understand the determinants of HR attributions, we use insights from social information processing theory. This theory argues that people make use of cues in their social environment in order to interpret certain events or situations (Salancik and Pfeffer, 1978). As such, we propose to take a social perspective, and focus on the cues that are obtained from other actors in the work environment as predictors of employee HR attributions. In the context of HRM and HR attributions, two important types of actors are expected to provide crucial information regarding HR practices in the work environment: line managers and coworkers. Given the devolvement of HR responsibilities to the line (Bos-Nehles et al., 2013; Nishii and Paluch, 2018), line managers are of crucial importance for the translation of HRM policies to the work floor (Purcell and Hutchinson, 2007). In line with recommendations of Nishii et al. (2008) and more recently of Hewett et al. (2018), we examine the attributions that the line manager communicates and their influence on employee attributions of HR practices.

In addition to line managers, coworkers can play an important role in the sensemaking process of employees as well. While in the HRM literature the role of coworkers has received only limited attention (with the exception of Jiang et al., 2017), other streams of literature have acknowledged the important role of coworkers, by for example studying the crossover of well-being related states (e.g., Bakker and Xanthopoulou, 2009) and through the notion of group affect (Walter and Bruch, 2008). Based on this it is argued that employees in dyadic work relationships will influence each other’s thoughts, emotions, and behaviors (e.g., Sherony and Green, 2002; Tims et al., 2015; Bakker et al., 2016) and at the same time their thoughts, emotions, and behaviors are influenced by the work context (e.g., the line manager) which they share (e.g., Fulmer and Ostroff, 2016). More specifically, by making use of social information processing theory it is argued that line managers and coworkers will exchange information and as a result a crossover of interpretations of certain work practices (i.e., HR attributions) will emerge. Furthermore, in line with Jiang et al. (2017) who studied the effect of surface-level demographic similarity, it can be expected that coworkers with deep-level similarity in terms of their work-related needs and motivations will tend to share their interpretations of HR practices more strongly. Our study thus builds on and extends this work by Jiang et al. (2017) by focusing on deep-level similarity in terms of similarity in work-related motivations in the context of HR attributions.

The contribution of the present study is threefold. Firstly, we shed more light on the predictors of HR attributions. While previous studies have shown that employees’ interpretations of why HR practices are used can have important effects on outcomes, we study whether line manager reports of why HR practices are implemented are predictive of HR attributions by employees. Secondly, the social context in which HR attributions are formed is studied in more detail by examining to what extent coworkers mutually influence each other’s HR attributions. Finally, insight is gained in the conditions under which coworkers more strongly influence one another by studying whether similarity in work motivations strengthens this process of mutual influence.

### Theoretical Background

#### HR Attributions

The notion of HR attributions can be understood in the stream of research which has focused on the relationship between HRM and performance. Within this area of research more emphasis has been placed on the role of employee perceptions of HR practices in understanding the relationships with outcomes (e.g., Gould-Williams and Davies, 2005; Purcell and Kinnie, 2007; Nishii and Wright, 2008). HR attributions examine employees’ explanations for why practices are used, which are referred to by Hewett et al. (2018) as “attributions of intent.” These attributions can be viewed as a fusion of attribution theory by Heider (1958) and attributional theory by Weiner (1979) (Hewett et al., 2018). Attributions in this area are distinguished in internal intent (i.e., under the control of the organization; HR practices aimed at attracting and retaining employees) and external intent (i.e., out of the organization’s control; HR practices aimed at complying with external forces such as legislation), with specific subdivisions for both internal and external intent (Hewett et al., 2018).

Building on these attribution theories and on previous empirical work (e.g., Koys, 1988, 1991; Bacon and Blyton, 2005), Nishii et al. (2008) have developed the concept of HR attributions. Drawing on social attribution theory they propose that employees can interpret HR related information differently based on the different meanings they attach to social stimuli. Furthermore, they distinguish internal and external attributions, with two internal attributions focusing on the perceived aim of management to increase service quality and employee well-being (so-called commitment-focused HR attributions), and two focused on reducing costs and exploiting employees (so-called control-focused HR attributions). The external attribution focuses on complying with union requirements (Nishii et al., 2008).

Previous studies have shown that these HR attributions are related to important outcomes, such as Organizational Citizenship Behavior (OCB; Nishii et al., 2008), turnover intentions, task performance (Chen and Wang, 2014), commitment, job strain and emotional exhaustion (Koys, 1991; Fontinha et al., 2012; Van de Voorde and Beijer, 2015; Shantz et al., 2016), in which commitment-focused attributions are generally positively related to desirable outcomes while control-focused attributions are generally related to undesirable outcomes (Hewett et al., 2018). Given the important outcomes of HR attributions, the current study focuses on, in line with recommendations of Hewett et al. (2018), the influence of the social context on HR attributions.

#### The Role of the Line Manager

Various studies have emphasized the important role of the line manager in the implementation of HR practices. The study by Nishii and Wright (2008) has been particularly important by emphasizing the difference between intended, implemented, and perceived HR practices. As a response to this contribution, several studies have examined the relationship between managerial reports of implemented HR practices and employee perceptions of these practices (e.g., Takeuchi et al., 2007; Liao et al., 2009; Den Hartog et al., 2013).

Drawing on social information processing theory (Salancik and Pfeffer, 1978), it can be argued that employees will make use of information provided by the line manager in order to understand organizational intentions. Line managers do not simply implement HR practices but they have a crucial role in providing employees with information that helps them to understand why specific HR practices are used in their team (Nishii and Paluch, 2018). Their communication is especially effective when they communicate HR intentions in a way that is distinctive, consistent and high in consensus, as this enables employees to consistently understand and respond appropriately to the HRM system (Bowen and Ostroff, 2004). The line manager thus plays an important role in optimizing these conditions in order to deliver clear HR signals to employees.

Based on the above, it is argued that employees understand and interpret HR practices based on the signals they receive from their line manager regarding why HR practices are implemented.

Hypothesis 1: Line manager HR attributions are positively related to employee HR attributions.

#### The Role of Coworkers

In addition to the effect of the line managers’ HR attributions on employees’ HR attributions, it is expected that coworkers will mutually influence each other’s HR attributions. The crossover of more cognitive appraisals of the work environment and particularly perceptions of HR practices has received less attention compared to the crossover of well-being related states (e.g., Westman, 2006; Bakker and Xanthopoulou, 2009). One exception is the study of Jiang et al. (2017) which has shown that employee perceptions of HR practices are influenced by perceptions of coworkers.

The theoretical explanation for this relationship between coworker perceptions can also be based on social information processing theory (Salancik and Pfeffer, 1978). Namely, in addition to line managers, coworkers are an important source of information regarding why HR practices are used. This means that coworkers will express their views on why certain HR practices are in use by the organization, which will influence the sensemaking process of the focal employee (Chen et al., 2013). The immediate social environment thus provides cues based on which employees’ can form or adapt their own interpretations and beliefs (Pollock et al., 2000).

In addition, coworkers can focus employees’ attention to certain information, making that information more salient. In turn, those more salient dimensions can then affect employee perceptions (Salancik and Pfeffer, 1978). Applied to the context of HR attributions, this means that when coworkers express a particular explanation of why HR practices are used, this information becomes more salient and can affect the HR attribution of the focal employee. Finally, Jiang et al. (2017) have argued that employees can also derive information from observing how HR practices are applied to coworkers. These observations can in turn be shared with coworkers and this process of information exchange can shape and alter HR attributions of both employees.

Based on the above, it is argued that coworkers represent the immediate social environment of employees, and employees will make use of information provided by these coworkers to make sense of why HR practices are in use. As a result, employees will be influenced by interpretations of HR practices formed by coworkers, and consequently HR attributions of coworkers are expected to be positively associated.

Hypothesis 2: Coworker’s HR attributions are positively related with each other.

#### Work Motivations of Coworkers

In addition to studying the role of line managers and coworkers, it is also important to understand the conditions under which employees are more likely to share the same view. Jiang et al. (2017) have previously shown that demographic similarity between the focal employee and coworker strengthens the relationship between their HR perceptions. These authors argued that “people with high demographic similarity may have similar work-related needs and motivations, and therefore are more likely to search for similar HR practices, which may set a common basis for them to perceive and interpret HR practices” (p. 6). In order to assess more directly whether similarity in work motivations indeed plays a role in this relationship as suggested by Jiang et al. (2017), we examine the role of work-related motivations in understanding the mutual influence of HR attributions between coworkers. Work motivations are defined as “the (un)conscious importance that workers attach to job characteristics and work outcomes” (Kooij et al., 2013, p. 90). Kooij et al. (2013) distinguish four motivations: (1) growth or development motivations which refer to job characteristics that enable making progress and using one’s talents, (2) esteem motivations which are job characteristics that concern prestige, status and promotion, (3) generativity motivations which refer to sharing knowledge and teaching younger generations, and (4) security motivations which concern the preference of protecting job security, pay and working conditions. As the latter work motivation is more or less taken for granted in the European employment relationship (Boselie et al., 2001), we focus on the work motivations growth and development, esteem, and generativity.

When employees share the same work motivations, it is expected that they will perceive the information of the coworker as more relevant for them (Jiang et al., 2017). This is in line with social information processing theory which argues that “the more similar someone is, the more relevant his or her views for understanding one’s own world” (Salancik and Pfeffer, 1978, p. 228). When the information obtained via the coworker is perceived as more relevant and more important, this will then influence their HR attributions more strongly. Also, employees will tend to have more interactions with coworkers that are more similar to them, as well as share and exchange more information with these coworkers that are more alike (Jiang et al., 2017). As a result, employees with similar work motivations will share more similar interpretations of why HR practices are used than employees that differ on their work motivations.

In addition, work motivations can be expected to influence employees’ HRM related preferences as particular HR philosophies and practices will support particular work motivations more strongly than others. For example, workers with growth and development motivations will be more focused on HR practices that emphasize that the organization values development of their workers whereas workers with esteem motives will feel more supported by HR practices related to reward and pay. Employees are thus expected to search for HR practices and more general signals (including the reasons why certain HR practices are implemented) that fit their work motivations (cf. Jiang et al., 2017). As a result, when employees share similar work motivations, they will search for and pay attention to similar practices and signals. When these search processes are shared, coworkers can confirm and reinforce interpretations of the colleague, and as a result coworkers will create shared interpretations of why HR practices are used. This effect will be stronger when coworkers have similar rather than different work motivations.

Based on the above it is argued that coworkers who are more similar in terms of their work motivations not only value the information obtained from the coworker more highly, but also view this information as more relevant, resulting in a stronger association between coworkers HR attributions as compared to when coworkers are less similar in terms of work motivations. This results in the following hypothesis:

Hypothesis 3: The similarity between coworker’s HR attributions will be stronger when similarity in work motivations (development and growth, esteem, and generativity) of coworkers is high rather than low.

## Materials and Methods

### Study Context

Data is collected in The Netherlands among triads of one line manager and two employees who work in the same organization. To study their mutual influence, employees needed to be based in the same organization and exposed to the same practices. However, to enable variation in scores on HR attributions, these triads were based in multiple organizations and respondents worked in a variety of sectors including both profit and non-profit.

### Procedure and Sample

Data collection is performed in collaboration with three master students (as part of their master thesis trajectory) under close supervision of the authors. Multiple studies have collected data via this procedure, among others, Bakker and Demerouti (2009) and Tims et al. (2015). Moreover, Demerouti and Rispens (2014) argued that this form of network sampling results in a heterogenous sample that facilitates generalization. Students have contacted employees in their own personal networks and were instructed to ask respondents to participate in the study by filling out a digital survey. In case the respondent contacted by the researcher was the line manager of a team, they were instructed to distribute the survey among two of their direct subordinates. Respondents were asked to fill in an anonymous code in the digital survey which was provided in the invitation email in order to enable matching of coworkers and their line managers.

Strict instructions were provided to line managers to select two direct subordinates who both belong to the same job group and who work closely together on a daily basis. In case the respondent contacted by the researcher was not in a management position, the respondent was instructed to invite both their direct supervisor as well as a coworker in the same job group with whom they worked closely on a daily basis to participate simultaneously in the study. It was important that coworkers had the same supervisor as well as the same job group as these coworkers are then almost certainly covered by the same HR policies and practices (Kehoe and Wright, 2013). Also, employees needed to be in close proximity to one another so that there were many opportunities to interact regarding HRM. Sharing these common features is necessary for being able to study mutual influence between coworkers.

For this study we made use of surveys. Respondents were provided with a link to an electronic questionnaire. Before the start of the survey, respondents were informed about the study and through this informed consent was obtained. Respondents were informed about the topic of the questionnaire. Also, they were informed that their data was anonymous and that confidentiality was ensured. In addition, respondents were provided with contact details in case they had additional questions. Respondents were informed that participation was voluntary and that they could end their participation at any stage. No sensitive topics or topics related to personal privacy, moral and/or ethical themes were addressed in the survey. All respondents were over 16 years old. The procedures used in this study are in line with Research Ethics and Regulations of the School of Business and Economics of the Vrije Universiteit Amsterdam and with national regulations. These regulations do not require written consent but consent was informed and respondents were required to click a button to start to the survey in case they decided to participate after being informed about the survey. For this study no approval from an ethical committee was asked as this type of survey is exempt from such approval in the Netherlands and by the institution leading this project.

A total of 325 complete questionnaires were collected. After deletion of unmatchable reports and incomplete triads containing either no managerial report or only one employee report (reflecting 20% of the 325 questionnaires), the sample consists of 87 triads (N line managers = 87, and N employees = 174). With respect to industry, 11.5% of our triads are working in the manufacturing industry, 17.2% in the healthcare industry, 13.8% in education and government, 32.2% in professional service industry, and 25.3% in the service industry. Of the line managers, 55.2% was male and the average age was 41.92 years (SD = 11.77). The majority of line managers holds a university degree (64.6%). Average organizational tenure of line managers is 10.11 years. For the employee sample, 45.4% was male and the average age was 33.18 years (SD = 11.26). The majority of employees hold a university degree (46.6%). Average organizational tenure of employees is 6.63 years. With respect to job group, our sample consists of 62 pairs of employees working in a (semi-) professional job (IT specialist, engineer), and 25 pairs of employees working in a non-professional job (sales employee, administrator).

### Measures

#### Employee HR Attributions

Employee HR attributions were assessed by asking why employees think that employees in their job group receive specific HR practices. Following Van de Voorde and Beijer (2015), five core HR practices central to HRM research were included in this study: Recruitment and selection, Training and development, Communication and participation, Performance management, and Reward. Following Nishii et al. (2008), employees were asked to rate for each practice to what extent this practice was implemented to (1) increase service quality, (2) increase employee well-being, (3) get the most work out of employees, (4) reduce costs, (5) and to comply with unions and/or law. This leaves open the possibility that one HR practice is implemented for multiple reasons. A total of 25 items was thus included to assess HR attributions. A five-point response scale was used ranging from (1) “strongly disagree” to (5) “strongly agree.” An example item is: ‘Employees in my job group are given the current development and career opportunities… in order to help employees deliver quality service to customers’. We conducted a confirmatory factor analysis to examine how well the data fitted the three-factor structure of HR attributions proposed by Nishii et al. (2008) containing a commitment-focused HR attribution (service quality and employee well-being HR attributions combined), control-focused HR attribution (cost reduction and exploiting employees HR attributions combined), and external attribution factor (union compliance HR attribution). The three-factor model fitted the employee data reasonably (χ^2^ = 452.58, df = 250; RMSEA = 0.068; CFI = 0.87). Following Nishii et al. (2008), Fontinha et al. (2012), and Chen and Wang (2014) we therefore combined items from the service quality and employee well-being attributions and combined items from the cost reduction and exploitation attribution, resulting in three HR attributions: commitment-focused HR attribution, control-focused HR attribution and union compliance HR attribution. Reliability of the commitment-focused HR attribution was 0.85, control-focused HR attribution 0.83, and union compliance HR attribution 0.80.

#### Line Manager HR Attributions

Line manager HR attributions were assessed in an identical manner to employee HR attributions but managers were asked regarding the goals and intentions behind the use of HR practices. An example item is “Employees that I supervise are given the current development and career opportunities… in order to help employees deliver quality service to customers.” The three-factor model fitted the managerial data reasonably (χ^2^ = 375.90, df = 262; RMSEA = 0.071; CFI = 0.86). Reliability of the commitment-focused HR attribution was 0.88, control-focused HR attribution 0.82, and union compliance HR attribution 0.83.

#### Work Motivation Similarity

Work motivation similarity is measured with 10 items that asked employees to indicate the importance they attached to certain job features or work outcomes that can be part of a job (Kooij et al., 2013). Answer alternatives ranged from (1) “totally not important” to (7) “very important.” Three work motivations were relevant to the current study: growth motives (four items, α = 0.86), esteem motives (three items, α = 0.78), and generativity motives (three items, α = 0.79). The hypothesized three-factor model fitted the data well (χ^2^ = 80.45, df = 31; RMSEA = 0.096; CFI = 0.94). An example item for growth motives is how much importance the employee attaches to “fully using my skills and abilities,” for esteem motives the importance attached to “prestige and status inside the company” and for generativity motives the importance attached to “the chance to teach and train others.” To measure employee similarity to their coworker we created two groups for each motivation type (one group scoring the same on the work motive, and the other group scoring dissimilar).

### Analytical Strategy

Because of our triadic data structure, the data are not independent. We therefore analyzed our data following the Actor-Partner Interdependence Model (APIM), specifically with the mutual-influence model (Kenny et al., 2006). The APIM was designed to deal with violations of statistical interdependence by including the coworker dyad as the highest unit of analysis, with employees nested within the coworker dyad. The mutual-influence model suggests that outcomes of the two members of a coworker dyad directly influence each other (i.e., reciprocal influence). Because the dyad members cannot be distinguished from each other based on some grouping variable (e.g., gender in heterosexual couples), the members of a dyad were treated as indistinguishable (Kenny et al., 2006).

In our dataset coworkers are nested within dyads (Level 2; *N* = 87 dyads, Level 1; *N* = 174 employees). We used multilevel analysis to test our hypotheses and used one-tailed significance tests. We controlled for organizational tenure, as tenure might influence the development of HR attributions (Nishii et al., 2008).^1^ Employee HR attributions and organizational tenure represented person (Level 1) data, while line manager HR attributions constituted a dyad-level variable.

## Results

Before testing our hypotheses, we examined whether the employee HR attributions differed between the coworker dyads. Partitioning the total variance into within- and between-dyad variance showed that 54.9% of the total variance of the commitment-focused HR attribution was attributable to between-dyad variation, and 33.2% of the total variance of the control-focused HR attribution was attributable to between-dyad variation, and 27.6% of the total variance of union compliance HR attribution was attributable to between-dyad variation.

Table 1 presents the descriptive statistics and correlations for the variables in our study. As shown in Table 1, line manager commitment-focused HR attribution and union compliance HR attribution is positively associated with employee commitment-focused HR attribution (*r* = 0.40, *p* < 0.05) and union compliance HR attributions, respectively (*r* = 0.45, *p* < 0.05). Line manager control-focused HR attribution was not significantly associated with employee control-focused HR attribution.

**Table 1:** Means, standard deviations, and correlations (*N* = 87 triads, 87 line managers, 174 employees).

Variable	Mean	SD	1.	2.	3.	4.	5.	6.	7.	8.	9.
1. Tenure - _employee_	6.63	7.83									
2. Commitment-focused HR attribution - _employee_	3.72	0.47	−0.15								
3. Commitment-focused HR attribution - _manager_	4.07	0.51	−0.26^∗^	0.40^∗∗∗^							
4. Control-focused HR attribution - _employee_	3.01	0.48	0.06	−0.05	−0.22^∗^						
5. Control-focused HR attribution - _manager_	2.94	0.63	−0.19	−0.05	−0.00	0.20					
6. Union compliance HR attribution - _employee_	2.63	0.59	0.10	−0.29^∗∗^	−0.22^∗^	0.25^∗^	−0.01				
7. Union compliance HR attribution - _manager_	2.24	0.76	0.03	−0.14	−0.10	0.22^∗^	0.02	0.45^∗∗∗^			
8. Growth motives similarity- _employee_	0.21	0.41	−0.08	−0.02	0.14	−0.14	−0.20	−0.11	0.04		
9. Esteem motives similarity - _employee_	0.17	0.38	0.08	0.02	0.11	0.05	−0.09	0.02	−0.05	0.14	
10. Generativity motives similarity - _employee_	0.16	0.37	0.04	0.13	0.08	0.03	0.03	−0.02	0.34^∗∗^	0.09^∗^	0.21^∗^

*^∗^p < 0.05, ^∗∗^p < 0.01, ^∗∗∗^p < 0.001.*

Table 2 displays the results of our multilevel analyses examining the influence of line manager HR attributions on employee HR attributions. In support of Hypothesis 1, line manager HR attributions were positively related to employee HR attributions (*B* = 0.35, *p* < 0.01 for commitment-focused HR attribution; *B* = 0.15, *p* < 0.05 for control-focused HR attribution; *B* = 0.35, *p* < 0.01 for union compliance HR attribution). Therefore Hypothesis 1 was supported.

**Table 2:** Multilevel estimates for predicting employee HR attributions.

	Commitment-focused HR attribution	Control-focused HR attribution	Union compliance HR attribution
Variable	Estimate	Estimate	Estimate
**Dyad-level predictors**			
Manager attribution	0.35^∗∗∗^	0.15^∗^	0.35^∗∗∗^
**Person-level predictors**			
Tenure	−0.00	−0.00	0.00
−2 log (lh)	241.02	310.41	377.21
Within-dyad variance	0.13	0.23	0.40
Between-dyad variance	0.12	0.11	0.09
Conditional ICC	0.49	0.33	0.18

*^∗^p < 0.05, ^∗∗∗^p < 0.001.*

Hypothesis 2 stated that coworker’s HR attributions are positively related with each other. For testing this hypothesis we calculated the conditional IntraClass Correlations (ICC)(1) using variance estimates from models including the control variable organizational tenure and the related HR attribution of the manager as predictors. ICC(1) measures the relation between the two outcomes when dyad members are indistinguishable. To test the significance of the conditional ICC(1) we estimated models with Level 2 random intercept and models without (see Hahn and Dormann, 2013 for a similar approach). The conditional ICC(1) for commitment-focused HR attribution was 0.49, and comparing the deviances of the models [Δ -2 log (lh) = 23.81, df = 1, *p* < 0.05] showed that the conditional ICC(1) was significant. The conditional ICC(1) for control-focused HR attribution was 0.33, and comparing the deviances of the models [Δ -2 log (lh) = 9.52, df = 1, *p* < 0.05] showed that this conditional ICC(1) was also significant. The conditional ICC(1) for union compliance HR attribution was 0.18, and comparing the deviances of the models [Δ -2 log (lh) = 2.68, df = 1, *p* > 0.05] showed that the conditional ICC(1) was not significant. These findings indicate that coworkers directly influence each other’s commitment-focused and control-focused HR attributions but not each other’s union compliance HR attribution. Therefore Hypothesis 2 was partly supported.

In Hypothesis 3, we predicted that the similarity between coworker’s HR attributions will be stronger when similarity in work motivations (development and growth, esteem, and generativity) of coworkers is high rather than low. Following the procedures described by Kenny et al. (2006), we calculated conditional ICC(1) for coworker dyads with similar work motives and coworker dyads with dissimilar work motives. Subsequently, to test whether the conditional ICC(1)’s differed between coworker dyads with similar and dissimilar work motives we conducted a Fisher *Z*-test. As shown in Table 3, in general we found that for coworker dyads with similar work motives the mutual influence of each other’s HR attributions was stronger compared to coworker dyads with dissimilar growth work motives (the conditional ICC’s of similar coworker dyads are higher than the conditional ICC’s of dissimilar coworker dyads). The Fisher *Z*-tests yielded significant results, indicating that the ICC are significantly stronger for coworker dyads with similar work motives, for four of the nine hypothesized interactions. In particular the *F*-test was significant for similarity in growth motives and commitment-focused HR attribution (*Z* = 2.28, *p* < 0.05), similarity in esteem motives and control-focused HR attributions (*Z* = 1.6, *p* = 0.055), and union compliance HR attributions (*Z* = 2.09, *p* < 0.05), and similarity in generativity motives and union compliance HR attributions (*Z* = 2.38, *p* < 0.05). As such Hypothesis 3 was partly supported.

**Table 3:** Conditional ICC values for similar and dissimilar work motivations to coworker.

	Growth motives			Esteem motives			Generativity motives		
Variable	Similar *N* = 18	Dissimilar *N* = 69	Fisher Z	Similar *N* = 15	Dissimilar *N* = 72	Fisher Z	Similar *N* = 14	Dissimilar *N* = 73	Fisher Z
Commitment-focused HR attribution	0.79	0.39	2.28^∗^	0.69	0.46	1.1	0.75	0.46	1.47
Control-focused HR attribution	0.53	0.31	0.98	0.64	0.26	1.6^#^	0.36	0.33	0.08
Union compliance HR attribution	0.26	0.17	0.32	0.63	0.09	2.09^∗^	0.70	0.09	2.38^∗∗^

*^∗^p < 0.05, ^∗∗^p < 0.01; ^#^p = 0.055. N refers to the number of coworker dyads.*

## Discussion

The current study has examined, based on social information processing theory (Salancik and Pfeffer, 1978), to what extent managers and coworkers influence HR attributions of employees. First, following recommendations of Nishii et al. (2008) and Hewett et al. (2018), the role of line managers in understanding employee HR attributions is examined. We have shown that line manager HR attributions are positively related to employee HR attributions. This relationship is stronger for the commitment-focused HR attribution and union compliance HR attribution than the control-focused HR attribution. This suggests that employees are more open to signals of the line manager which connote positive consequences for employees rather than signals of the line manager which connote lower levels of concern for employees and a more cost-driven control focus. In addition, it is shown that coworkers mutually influence each other’s HR attributions, controlled for HR attributions of the line manager.

This study has also shown that similarity in work motivations matters for the extent to which coworkers influence each other’s HR attributions. We found consistent support for the idea that coworkers influence each other’s HR attributions more strongly when similarity in work motivations is high rather than low. This is also the case for the union compliance HR attribution as we found that employees did not influence each other’s union compliance HR attribution directly while the relationship was present when employees were more similar in terms of their work motivations (i.e., for esteem and generativity motives). In line with social information processing theory, colleagues with similar work motivations seem to value the information of this colleague more highly and as more relevant than that of colleagues with dissimilar work motivations, resulting in HR attributions that are more similar (Salancik and Pfeffer, 1978; Jiang et al., 2017).

Our results also suggest that a match between the content of the HR attribution and the type of work motivation plays a role as similarity in esteem motives strengthened the sharing of control-focused HR attributions and similarity in growth motives strengthened the sharing of commitment-focused HR attributions. This suggests that employees search for signals that match their preferences; coworkers who both strive for esteem and status will interpret HR signals in a performance-focused manner, while coworkers both striving for growth and development search for signals that confirm that the organization values employee wellbeing and development. It should be noted that we not only found that employees who are more similar in their work motivations tended to share certain HR attributions more strongly than more dissimilar coworkers, but also that when work motivations were similar, coworkers consistently scored high on the work motivations that they shared. In other words, in our sample coworkers did not share low scores on work motivations. This suggests that not only the match between the type of work motivations and the type of attribution matters but also the direction of the work motivations (i.e., high versus low absolute score). This is consistent with findings in the climate literature in which strong climates are frequently characterized by high strength and also high absolute levels, meaning that coworkers simultaneously score high on the constructs (Lindell and Brandt, 2000; González-Romá et al., 2002). In the current study this might be caused by mechanisms related to the attraction-selection-attrition framework (Schneider, 1987), which over time results in a workforce that values and shares those specific aspects that the organization also values.

These findings contribute to practice as they shed more light on how employees make sense of their work environment. It confirms the important role of line managers but also underlines the role of coworkers in shaping employees’ HR attributions. This implication is important as we do know that HR attributions influence employees’ attitudes and behaviors at work (Hewett et al., 2018). As coworkers can influence each other’s interpretations of why HR practices are used, it might mean that not all messages need to be communicated directly to each worker by the line manager but that these messages might also be spread by coworkers themselves. In the current study, however, it is possible that coworkers spread interpretations about HR practices that are inconsistent with the intended goal of management. Our results indicate that it is therefore important to inform both the line manager and coworker extensively but to also pay attention to the specific work motivations of different employees in one team. Also, future studies might look into the conditions under which coworkers are more susceptible to the influence of potential inconsistent messages (Westman and Bakker, 2008). This might for example depend on HRM system strength, with employees in strong HRM systems being less likely to be influenced by inconsistent messages (Li et al., 2011).

### Limitations

While this study adds to the current literature on HR attributions by examining the role of line managers and coworkers in HR attributions of employees, several limitations of the study can be identified. First, the current study required a specific combination of respondents which has complicated the data collection process. More specifically, respondents needed to be interrelated as it was required to collect data from two closely connected coworkers as well as their line manager. As it is more difficult to collect this type of data, sample size is limited which has implications for the statistical power of the study.

Second, the study is cross-sectional in nature which means that no causal inferences can be made. It would be informative if future studies would examine the process of influence between coworkers and their line manager over time. It could for example be examined how long it takes for HR attributions of employees to affect HR attributions of others. In addition, specific critical events which might trigger sensemaking and interaction between colleagues regarding why HR activities are used could be studied to gain more insight in how HR attributions change over time.

Third, given the complex triadic data structure that was required (line manager-employee-coworker), we were unable to randomly select coworkers. The line managers selected two employees or the coworkers themselves selected a colleague for participation in the study. The coworkers were required to stem from the same job group and to work together on a daily basis. While this procedure has been used in similar previous studies (e.g., Bakker and Xanthopoulou, 2009; Tims et al., 2015), and Demerouti and Rispens (2014) argue that using students to recruit participants may be helpful with difficult study designs, it is unclear whether a selection bias by the line manager or mutual liking of coworkers could have potentially affected the results.

Finally, as the current study aimed to examine how the social context affected HR attributions of coworkers, other sources of information that workers use to understand why HR activities are used might have been omitted, such as policy documents, newsletters, and information distributed via e-HRM systems. Future studies might look into whether different sources of information are valued and shared differently and could examine the role of consistency of information within and across these different sources (cf. Den Hartog et al., 2013). However, given that we aimed to take an interpersonal perspective on HR attributions these types of data sources did not fit the current study.

### Future Research

As the current study shows that line managers and coworkers influence employees’ interpretations of the work environment, future studies could make use of similar triadic and/or dyadic study designs in order to examine how coworkers influence each other in terms of their perceptions of HR activities and the work environment more generally. This interpersonal perspective could add to the HRM literature as it might enable a better understanding of how HR perceptions are created and gives insight in what role interpersonal interactions and information exchanges between coworkers play in this. Also, as our study has shown that more similar coworkers share HR attributions more strongly, future studies could look into the mechanisms that might explain this (Sanders et al., 2014). As similarity could affect frequency of communication and information exchange, these mechanisms could be studied further (Westman and Bakker, 2008). In addition, the perceived relevance and trustworthiness of information received from both coworkers and line managers might also be examined in more detail.

To further understand these processes of influence between coworkers it might also be important to study personality factors such as empathy (e.g., Bakker and Demerouti, 2009) as it would be expected that workers who score high on empathy will be more sensitive to interpretations of coworkers which will thus strengthen the influence of colleagues on the focal employee. Another personal factor that is examined in social information processing research is self-monitoring. Self-monitoring refers to a personality characteristic that indicates a sensitivity toward information send by others (Gangestad and Snyder, 2000). Workers with high levels of self-monitoring thus pay attention to what others in the work environment communicate to them and as such may be more likely to be influenced by coworkers in their attributions of HR practices.

In addition to personality characteristics of coworkers other characteristics of the work environment or work context might also play a role such as the strength of the HRM system (Bowen and Ostroff, 2004). It could be argued that when system strength is low, this creates stronger interactions between coworkers in order to make sense of their environment (Ostroff and Bowen, 2016). Reversely, when system strength is high, there might be a reduced need for information seeking and exchange (Ostroff and Bowen, 2016). The influence of strength of the HRM system and the role of the line manager therein thus requires further investigation (Li et al., 2011; Sanders et al., 2014; Hewett et al., 2018).

## Conclusion

All in all, the current study contributes to existing literature in two ways. Firstly, this study is the first to show that managerial reports of HR attributions are related to employee reports of HR attributions. This is critical as it underlines the important role of line managers in creating HR related perceptions of employees. Explicitly asking managers to report why they implement HR practices for workers in their team, instead of why managers think they themselves receive certain HR practices, results in more accurate information as the line manager and employee rate the same HR practices. Secondly, the current study examines employees in coworker dyads. While literature (Pollock et al., 2000; Chen et al., 2013) has shown the important role that coworkers have in influencing others, this study is the first to apply this interpersonal perspective to the literature on HR attributions. We showed the influence of coworkers on employees’ HR attributions, and investigated similarity in work motives as a boundary condition of this mutual influence. Taken together, our findings support our proposition that employees’ social environment at work, particularly their line manager and coworker, matter in HR attribution processes. The findings stress the need to consider the social environment at work to more fully understand the factors that create employees’ understandings of HR practices.

## Author Contributions

SB: theorizing, data collection, writing, and coordinating. KVDV: theorizing, data collection, data analysis, and writing. MT: theorizing, data analysis, and writing.

## Conflict of Interest Statement

The authors declare that the research was conducted in the absence of any commercial or financial relationships that could be construed as a potential conflict of interest.

## Footnotes

1 Additional analyses were performed in which industry and job group were included as control variables. As this did not change the pattern of results, we report the findings without the inclusion of these additional control variables.
